# Visualizing Parcel-Level Lead Risk Using an Exterior Housing-Based Index

**DOI:** 10.3390/ijerph22010016

**Published:** 2024-12-27

**Authors:** Neal J. Wilson, Ryan Allenbrand, Elizabeth Friedman, Kevin Kennedy, Amy Roberts, Stephen Simon

**Affiliations:** 1 Center for Economic Information, University of Missouri-Kansas City, 5120 Rockhill Rd, Kansas City, MO 64110, USA; 2 Environmental Health Program, Children’s Mercy Kansas City, 2401 Gillham Road, Kansas City, MO 64108, USA; rnallenbrand@cmh.edu (R.A.); efriedman@cmh.edu (E.F.); 3 Healthy Indoors Training and Consulting, LLC., Lawrence, KS 66044, USA; kkennedy740@gmail.com; 4 Kansas City, Missouri Health Department, 2400 Troost Ave., Kansas City, MO 64106, USA; amy.roberts@kcmo.org; 5 Department of Biomedical and Health Informatics, University of Missouri-Kansas City, 2411 Holmes St, Kansas City, MO 64108, USA; simons@umkc.edu

**Keywords:** childhood blood lead levels, lead hazard risk, housing, social determinates of health, environmental public health, place-based approach

## Abstract

Pediatric lead poisoning remains a persistent public health problem. Children in the US spend the preponderance of their time at home; thus, housing is an important social determinant of health. Improving health outcomes derived from housing-based sources involves differentiating the risks posed by the existing housing stock. In this paper, we developed a parcel-level lead risk index (LRI) based on external housing conditions and the year of home construction. The purpose of this study was to introduce a housing-based lead risk index (LRI), developed using retrospective data, to estimate parcel-by-parcel variation in housing-based lead risk. We described how the LRI is constructed, relate it to the likelihood of a pediatric occupant’s blood lead level (BLL) > 3.5 µg/dL using Lasso regression (*n* = 6589), visualized this relationship graphically, and mapped the outcome. We found that mapping the LRI provided more information at a more precise geographic level than was possible using other public health surveillance methods.

## 1. Introduction

Lead in the built environment is a significant public health hazard [1,2]. Although lead is a naturally occurring element, the large majority of human exposure to it is from anthropogenic sources [3,4]. The regulation of lead in industrial products is recognized as a major public health victory [5]. However, lead persists in the built environment, and children continue to be subjected to unsafe levels of exposure [6,7]. The primary sources of childhood lead exposure in the US are housing (deteriorating lead paint [8,9], water service lines [10,11]), soil (from legacy pollution from leaded gasoline [12,13], and industry [14]), ongoing industrial point sources (factories, power plants, and aviation [15]), and consumer products [16]. Pediatric exposure to lead is associated with adverse social and health impacts, including but not limited to negative behavioral [17], socio-economic [18,19], neurological [20], renal [21], endocrine [22], cardiovascular [23], and developmental effects [24].

The burden of lead exposure is not borne equally in our stratified society. The longitudinal data collected for the National Health and Nutrition Examination Survey (NHANES) indicates the percentage of US children with blood lead levels (BLLs) greater than 5 µg/dL is consistently higher for Non-Hispanic Black children when compared to the Non-Hispanic White cohort [25]. Those households with a family income-to-poverty ratio of less than 1.3 have consistently higher BLL than the more affluent members of their cohort [26]. The continuing impact of racist and classist housing policies in the US is manifest in geographic concentrations of lead poisoning [15,27]. Disparities in lead exposure, apparent at the household level, are also observed at the larger geographic areas of zip code and census block group levels [28,29].

The EPA’s EJScreen is an environmental mapping and screening tool that quantifies patterns of lead risk exposure [30,31]. Other similar indices focused on lead risk also exist, such as the Schultz Model and the US Department of Housing and Urban Development’s lead-based paint index [32,33]. The criteria used by these tools to identify lead risk fit broadly into the categories of environmental (proximity to traffic and pollution sources [25], air quality issues like PM 2.5 and ozone, water quality issues like proximity to wastewater drainage) and socioeconomic indicators (age of housing and housing condition [25], percent racial and ethnic population, and income level [25,28,34,35,36], population age education level [35]). These criteria data are gathered for a screening-level overview and presented at aggregate levels of geographic generality (census block groups, census tracts, zip codes, neighborhoods).

Housing-level geography is relevant for developing individually focused intervention strategies to prevent pediatric lead poisoning [37,38]. Children—particularly toddlers—spend the majority of their time in the home [39], and it is in the home where children are likely to be exposed to lead [40]. Housing has a profound effect on health. The National Academy of Sciences has recommended conceptualizing housing as one of the social determinants of health [41]. Housing-based social determinants, such as the presence of lead hazards inside the home and in the soil around the homes, are primarily a function of the year of home construction and reported at the neighborhood level [26,41,42]. However, the focus on neighborhood-level indicators (or other aggregate geography) for health interventions can reinforce an ecological fallacy by assuming away variation within the geography [43]. Though an entire neighborhood may be of roughly the same age, that does not imply that all homes pose an equal lead risk to their residents. Similarly, all residents of a racially homogenous neighborhood are not necessarily at equal risk for lead exposure [44,45]. House-by-house variation in housing quality, deferred maintenance, and building materials can differentiate lead risk at the household level.

Our work embodied a low-cost and non-invasive housing-focused lead risk identification method that can be effectively presented in maps. Mapping to mitigate lead exposure and exposure disparities is a priority of the federal action plan to eliminate lead poisoning [46]. Proactive identification of housing-based lead risk enables the prevention of pediatric exposure without blood lead surveillance data and before it has the chance to disrupt a child’s life [36,47]. The objectives motivating this research are (i) to develop a low-cost, non-invasive, geographically precise index of housing-based lead risk based on publicly available information and (ii) to illustrate the usefulness of the index visually. An associated challenge of constructing our exterior lead risk index (LRI) is the collection of reliable observations of exterior housing condition indicators to include in the index. The animating goal of this research is to add a predictive lead risk indicator to the arsenal of public health surveillance methods.

## 2. Methods

This study introduced a housing-based lead risk index (LRI) developed to estimate parcel-by-parcel variation in housing-based lead risk. The LRI is generated by the interaction of two publicly available data sources (year of home construction and exterior housing condition). We trained our LRI retrospectively on associations between parcel-level variation in exterior housing conditions and address-level patient blood lead observations. This study builds on previously published research on associating various exterior housing conditions generated from the Center for Economic Information’s Neighborhood Housing Conditions Survey (NHCS) to predict elevated pediatric blood lead levels [48].

### 2.1. Data Sources

The Kansas City, Missouri Health Department provided de-identified patient-level blood lead observations (IRB #11120500). This dataset was queried from a database of all blood lead tests between January 2000 and December 2013 in the state of Missouri. The lead dataset includes information regarding patient address, date of birth, sex, date of test, test type, and health department jurisdiction (*n* = 6589). The continuous ‘Test Result’ variable was transformed into a dichotomous variable, indicating the observed value is greater or less than the 3.5 µg/dL reference value [49]. The dichotomous ‘Test Before 2005’ variable was created to account for the secular decline in average BLL during the study period [50]. Two additional dichotomous variables were generated to indicate the age of the child at the time of the test. These variables reflect an established non-linear relationship between a child’s age and their BLL [51,52]. Each BLL observation was geocoded to their postal address and joined to observations of exterior housing conditions from the same calendar year. The process of associating the lead data with the housing conditions data is discussed in Methods S1 and Figure S1 (both in Supplemental Materials).

The NHCS was used to collect exterior housing conditions. Details about the history and implementation of this survey tool are available in other publications [53,54]. The NHCS was carried out by surveyors in person and used an objective rubric to guide the data collection process. The NHCS was designed to be low cost (~USD 8/parcel), gather public information, and provide more detail about the condition of the home than is generated by the municipal housing code. The NHCS surveyors assign an ordinal rating to each housing category (Exterior Paint, Windows and Doors, Porch, Roof, Foundation and Walls) which was used in conjunction with the year of home construction to calculate the LRI.

The year of home construction at the parcel level was provided by the Mid-America Regional Council, with supplemental detail provided by Architectural and Historical Research LLC. The year of home construction was transformed into a categorical variable which reflects two critical years in the history of the production and use of lead paint; 1952, when production of interior lead paint was ended, and 1978, when exterior lead paint was no longer available for use in residential applications [42]. The salience of these dates is reflected in the NHANES reporting on pediatric BLL [9,26].

### 2.2. The Exterior Lead Risk Index

The LRI is generated by combining the era of home construction with the five NHCS categories (Exterior Paint, Windows and Doors, Porch, Roof, Foundation and Walls). Table 1 describes the potential values for these variables. The LRI is based on our external neighborhood housing conditions survey, which uses an ordinal rating system to represent qualitative data. Thus, the LRI is also an ordinal variable. Equation (1) describes how the LRI is calculated.

(1) $${LRI}_{i}=\sum_{j=1}^{5}{era}_{i}*{condition}_{ij}$$

In this formula, *i* is the house in question and *j* is the housing category. The model is constructed to focus on variation in those homes that may pose an active lead risk to their inhabitants, it amplifies variation among older homes and homes with housing categories in disrepair. Conversely the model minimizes variation among homes built in 1978 or later and those with housing conditions rated as good or excellent. The lowest possible LRI is 0 and the highest is 30. Examples of how to calculate the LRI are provided in Methods S2 (in Supplemental Materials).

### 2.3. Statistical Methods

We used a logistic lasso regression to estimate the observational relationship between LRI values and the likelihood of childhood blood lead levels over 3.5 µg/dL [49]. We took this step to corroborate our intuition that rising LRI values are associated with an increased likelihood of elevated pediatric lead exposure. Lasso models are frequently used in environmental and health research [48,55,56]. The LRI was transformed into a contrast matrix to estimate a dose–response relationship between index score and elevated blood lead levels. We included, in our statistical analysis, the confounding variables for the age of the child and the year in which the blood lead test took place. We also included several infrastructure conditions, also transformed into a contrast matrix, that are unrelated to pathways of lead exposure (public walks, curbs, streetlights) as a sensitivity analysis to test if the model produced spurious correlations.

Lasso models are a ‘data mining’ technique, they perform an algorithmic selection of explanatory variables through a shrinkage operation on those regression coefficients that are close to zero. Our analysis added a non-negativity requirement as a logical condition on the coefficients. The lasso model does not produce confidence intervals, and it selects and estimates the group of variables among those given which have the largest impact on the dependent variable. It estimates a range of shrinkage parameters (essentially lower limits that the coefficient must exceed to be included in the regression) and calculates the binomial deviance produced by the model for each parameter. This estimation of multiple shrinkage parameters reflects the cross-validation process inherent to the lasso technique and is designed to avoid overfitting the data. The benefits of the lasso technique are accuracy and legibility at the cost of a potential increase in bias [57].

After we computed the LRI, we mapped the result at the parcel level. We reproduced a parcel-level lead risk map that uses the LRI next to a map that visualizes the standard parcel-level risk parameters.

Our statistical analysis was conducted using the R programming language (version 4.2.0) via the RStudio environment (2022.12.0 Build 353). We used the glmnet package (4.1-4) to compute our lasso regressions and the ggplot2 package (3.3.6) to create our graphs. The mapping was performed in ArcGIS pro 3.0.0.

## 3. Results

The population characteristics of the patient-level blood lead observations are reported in Table S1 (in Supplemental Materials). The state of Missouri did not collect race or ethnicity information about blood lead test recipients. The blood lead sample was 51% male. Venous blood lead exams constituted 62% of all tests, 7% of the exams were of unknown test type. The 6-to-17-month age range constituted 28% of the sample, the 18-to-36-month age range constituted 26% of the sample. The distribution of tests per year varies between 2000 and 2013; from a high of 21% in 2008 to as low as 0.7% in 2012. Thirty percent of the tests took place between 2000 and 2005. The distribution of NHCS scores are summarized in Table S2 and the location of homes is summarized in Table S3 (both tables in Supplementary Materials). 

Table 2 reproduces the lasso regression coefficient estimates for the shrinkage parameter that minimizes the model’s binomial deviance. The algorithmic variable selection process has eliminated several variables from the model (for more about this process, see Figure S2 in Supplemental Materials), we included these variables in Table 2, listing their coefficient estimate as a midline decimal point (‘·’). Several LRI contrast values (0–6 vs. 7, 0–10 vs. 11, etc.) are not listed due to their absence in the source data. The coefficient values presented in Table 2 are log-odds contrasts which require additional calculation to be easily interpretable. For more information about interpreting the log odds estimates for this lasso model, see Methods S3 (in Supplemental Materials). Table 2 indicates that the number of observations per LRI decreases as the index value increases.

**Figure 1:**
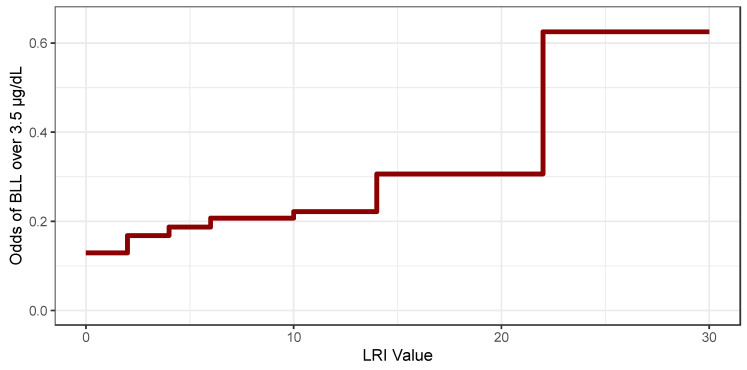
Interpreting LRI log odds contrasts.

Our model was structured to reflect a dose–response relationship between LRI and the odds of BLL greater than 3.5 µg/dL. Figure 1 illustrates this monotonic relationship graphically. The LRI was designed to incorporate only publicly available information; thus, we did not use any of the variables related to the individual patient in the construction of Figure 1. Child-focused information is not publicly available and will not be available when the LRI is used prospectively. As the LRI increases the odds of a child having a blood lead level over 3.5 µg/dL increases in a stepwise manner. Figure 1 shows small increases in the odds of a BLL > 3.5 µg/dL at low LRI levels and two larger jumps, the first at 14 as the LRI enters the mid-range and a second larger jump at an LRI of 22.

**Figure 2:**
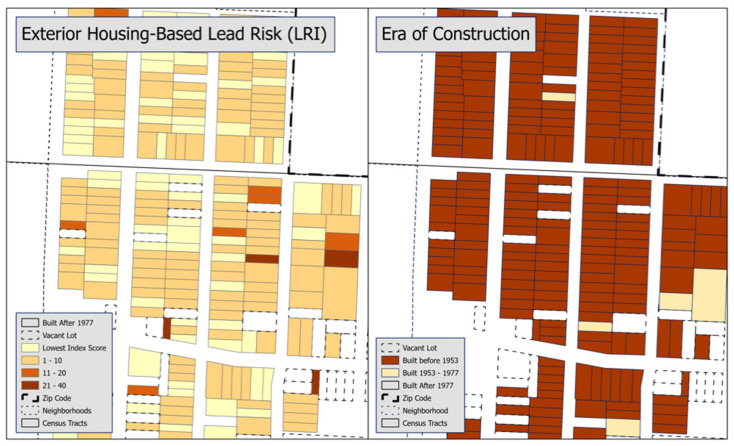
Contrasting maps of LRI and era of housing construction.

To illustrate the contrasting degree of detail provided by the LRI and traditional methods, we mapped lead risk for an area of Kansas City, Missouri. Figure 2 visualizes the LRI as a 5-level scale. The visualization categories in these maps are chosen with reference to Figure 1. There is considerably more variation in the map of exterior housing-based lead risk than is displayed in the era of construction map.

## 4. Discussion

The central focus of this paper is the introduction of an external housing condition-based lead risk index (LRI). Our research is built on several related ideas. Housing is an important social determinant of health [37,58]. Preventing environmental exposure to health hazards before they require a trip to a doctor’s office is a goal of public health [59]. The connection between housing and occupant health frames housing interventions as a form of health care [37,58]. There is long-standing public support for lead remediation. The US Department of Housing and Urban Development has been funding lead remediation activities since the passage of the 1992 Residential Lead-Based Paint Hazard Reduction Act. Our LRI is designed to actuate this framing of housing as healthcare for the geographic level at which children live.

Our statistical estimates are consistent with our intuition that increasing LRI values are associated with an increasing likelihood of a child having an elevated blood lead level. Figure 1 illustrates that the likelihood of elevated blood lead increases faster at higher LRI values. It is unclear from the results presented here what LRI value should trigger an intervention, considering there is no safe level of pediatric blood lead exposure [7].

Two common issues with the public remediation of lead in housing are limited funds available for remediation and residents’ hesitance to participate in public programs where their private residence receives remediation. The LRI is a tool that can help economize available funds. Our method calculated the LRI using publicly available information, the year of home construction, and exterior residential conditions. To gather exterior housing conditions, we used the CEI’s NHCS, a low-cost (approximately USD 8 per parcel) tool that was used in several peer-reviewed applications.

Neighborhood buy-in is essential to overcoming public resistance to participation in lead-remediation programs. The LRI cannot operate alone. Referrals remain the best recruitment tool available. Lead poisoning is a social problem, both in its causes and effects. Therefore, the eradication of lead poisoning entails collective action. Neighborhoods matter for implementing lead-abatement initiatives; indeed, there can be no effective public health strategy without community buy-in. Implementing the NHCS, direct observation from inside the neighborhood, can help organize the people in the neighborhood being surveyed. The LRI should be used to augment and enhance traditional recruitment and public health surveillance methods.

The LRI can be used to sharpen the focus within the geography of risk identified by approaches like the EJ Screen or the EPA’s Lead Exposures Hotspots Analysis [60]. The persistence of race- and class-based discrimination manifests (among other ways) as segregation into neighborhoods with housing-based lead hazards. The available tools for determining lead exposure hotspots use publicly available data to calculate lead-based risk. The geographies of risk identified by screening tools reflect social vulnerability and the maldistribution of political and economic power, and resources more generally across the built environment [15,61]. Race, class, and era of home construction are the primary variables used to define geographies of lead risk. However, neighborhoods are generally built at the same time, and because race and class are publicly available only at the aggregate level (i.e., Census tracts, neighborhoods, zip codes, etc.), these tools are not useful for differentiating lead risk on a house-by-house basis. The need for another tool to identify troubled homes within a hot spot is acknowledged on the EJScreen website, which states it “should be used for a ‘screening level’ look. Screening is a useful first step in understanding or highlighting locations that may be candidates for further review [31]”. The maps in Figure 2 illustrate the usefulness of the LRI for a parcel-level review. There are no homes in Figure 2 built after 1977. These maps contain two census tracts, both of which score in the 95–100 percentile of the EPA EJ screen’s Environmental Justice Index for lead paint. Together, this means, at the screening level, all these homes are identical. Our LRI is not useful for a screening level analysis, but it does add variation to an identified location. The LRI is intended to be used in conjunction with screening-level tools that consider race and income in their calculations; together, they will position public health workers to provide individually focused, housing-based, pre-emptive care in the communities most in need of support.

The modeled approach used to estimate the LRI’s relationship to blood lead levels has several characteristics in its favor. Our statistical method elides problems that follow from small and biased samples; it utilized a large dataset (n = 6589) derived from the complete population of blood lead tests from the 2000–2013 period in Kansas City, MO. The binomial lasso model did not generate spurious coefficients for the unrelated infrastructure conditions we included in the model. Mapping the LRI onto the source geography translates non-intrusive, publicly available observations of the built environment into a legible description of potential housing-based lead exposure risk to children.

The results we present in this article are retrospective. The predictive power of the LRI should be verified before it is used to identify homes for lead-safe interventions or other public health applications, its predictive power should be verified. The next step in this research agenda is the following test procedure: implementation of an NHCS, calculation and mapping of the LRI, neighborhood outreach on the basis of the LRI, and a comparison of lead risk inspection results with LRI values. If the LRI predictions are mirrored by interior lead observations, the LRI can be counted among the tools of public health surveillance. The salience of the uncertain geographic context problem to the LRI—where a study subject is geolocated, but the amount of time spent at the location is unclear [62]—can only be resolved with further study. An important and related issue is implementing effective techniques for focused recruitment and enrollment of residents in LRI-identified homes and the referral to programs that remediate lead hazards.

### Limitations

Our study has several limitations that should not be glossed over. The LRI was generated using retrospective data; it requires ground truthing with a new NHCS and blood lead observations to validate the associations described. The NHCS was created in reference to the building codes of Kansas City, Missouri; it is unclear whether a tool with categories based on these codes will be ideal in areas with substantially distinct housing stock. The NHCS and LRI may need to be revised to suit the housing stock of other areas of the US or in international cities. The methods used to gather NHCS data casts doubt on the applicability of the LRI, as articulated, in places where it is difficult to observe home conditions from the road (rural areas, cities where privacy hedges or walls are common.

Our exterior lead risk index does nothing to suggest solutions to racist/classist housing practices that sort the most vulnerable into the worst quality homes; it may, by improving the techniques of discovery, reward negligent landlords with public funds directed toward remediation. The LRI operates via the likelihood of elevated blood lead levels, yet never arrives at a position of certainty. There may exist important unobservable sources of lead risk such as waterlines, soil, point source pollution, or lead-contaminated consumer products (such as toys, bottles, and food utensils), which vary at the parcel level and, nonetheless, remain invisible to the LRI.

## 5. Conclusions

In just over twenty years, the Kansas City, Missouri Health Department has completed over three thousand lead-safe housing remediations. However, this achievement is dwarfed by the scale of the problem. The American Housing Survey estimates there are more than 156,000 homes in Kansas City from the era of lead paint, while nationwide, there are some 22 million homes with one or more significant lead-based paint hazards [63]. The LRI can help differentiate and prioritize these homes, helping public health professionals focus on those homes in greatest need of attention.

There are clear benefits to developing a method for proactively identifying lead risk at the parcel level, which is evidence-based, cost-effective, non-intrusive, and focused on a small geography [64,65]. Our exterior housing-based lead risk index formalized the intuition connecting housing conditions to pediatric blood lead levels, quantifying the association between housing quality and health. The LRI can be mapped at the address level and consulted to focus outreach programs. This process will enable customized intervention strategies better tailored to the precise locations where children are exposed to lead. With prospective testing, our method of using publicly available information to construct a housing-based lead risk index marks the LRI as a promising tool for developing empirically-based preventive care.

## Figures and Tables

**Table 1:** Era of construction and housing condition values.

Era of Home Construction	Value	Condition of Housing Category	Value
1978 or later	0	Excellent or Good	0
1953–1977	1	Substandard	1
1952 or before	2	Seriously Deteriorated	2
		Severely Deteriorated	3

**Table 2:** Lasso log odds coefficient estimates.

Variable	Observations	Lasso Coefficient Estimate	Variable	Observations	Lasso Coefficient Estimate
(Intercept)		−1.909			
**LRI Contrasts**			**Child-focused Variables**		
LRI 1	368	·	Test Before 2005	1983	1.52
LRI 2	1399	0.3109	18–36 months	1709	0.4067
LRI 3	36	·	37–72 months	2987	·
LRI 4	899	0.1289	**Public Walks Contrasts**		
LRI 5	21	·	Ordinal Rating 2	2162	·
LRI 6	548	0.127	Ordinal Rating 3	1082	·
LRI 8	488	·	Ordinal Rating 4	359	·
LRI 9	2	·	Ordinal Rating 5	512	·
LRI 10	205	0.0859	**Curbs Contrasts**		
LRI 12	88	·	Ordinal Rating 2	2545	·
LRI 14	60	0.4393	Ordinal Rating 3	1006	·
LRI 16	48	·	Ordinal Rating 4	699	·
LRI 18	16	·	Ordinal Rating 5	358	·
LRI 20	8	·	**Streetlights Contrast**		
LRI 22	7	1.3288	Ordinal Rating 2	300	·
LRI 24	1	·	Ordinal Rating 3	21	·
LRI 26	1	·	Ordinal Rating 4	2	·
LRI 30	1	·	Ordinal Rating 5	4	·

## Data Availability

Housing condition survey data are available upon request. Blood lead testing data contain private health information and are unavailable due to privacy restrictions.

## Supplementary Materials

The following supporting information can be downloaded at: https://www.mdpi.com/article/10.3390/ijerph22010016/s1; Methods S1: The process of associating the NHCS and blood lead observations; Methods S2: Calculating the lead risk index; Methods S3: Interpreting the lasso model; Table S1: Characteristics of lead testing data; Table S2: Characteristics of housing data; Table S3: Housing location by community district; Figure S1: Study sample selection process; Figure S2: Variable Selection Using Lasso Regression.
