# Supplementary material for: Visualizing Parcel-Level Lead Risk Using an Exterior Housing-Based Index
Source: Int J Environ Res Public Health. 2024 Dec 27;22(1):16. doi: 10.3390/ijerph22010016 (PMC11765214; doi:10.3390/ijerph22010016)
Supplement: Supplementary file 1 [file ijerph-22-00016-s001.zip › ijerph-3306907-supplementary.pdf]

Online Supplement

"Visualizing Parcel Level Lead Risk Using an Exterior Housing-Based Index"

Neal Wilson, Ryan Allenbrand, Elizabeth Freidman, Kevin Kennedy, Amy Roberts, Steve Simon

Methods S1: The Process of Associating the NHCS and Blood Lead Observations
Methods S2: Calculating the Lead Risk Index
Methods S3: Interpreting the Lasso Model
Table S1: Characteristics of Lead Testing Data
Table S2: Characteristics of Housing Data
Table S3: Housing Location by Community District
Figure S1: Study Sample Selection Process
Figure S2: Variable Selection Using Lasso Regression

Methods S1: The Process of Associating the NHCS and Blood Lead Observations

The blood lead observations provided by the Health Department and the NHCS observations were both geocoded in an address-matching process using the ArcGIS pro software. After geocoding, the blood lead data were filtered by age and spatially joined with those NHCS observations of their home address, which took place during the same calendar year. The following quotation is from a previous publication on associations between the data on which our study depends.

> Among 201,499 observations in the health department data, 171,305 of these were in the pediatric population aged 6–72 months. Of the pediatric population, 100,656 of the children were located within Kansas City, Missouri, with a valid address. Among the pediatric population with a valid address, 89,671 observations were within 100 m of a home surveyed by the Neighborhood Housing Conditions Survey (NHCS). The 100-m distance was selected to account for variation between the centerline geography used in the first stage of the address match procedure and the parcel geography used to complete the address match procedure. The geocoding process by which these selections were made are found in Wilson et al [46]. Of the observations within 100 m of a surveyed parcel, 11,763 observations were from the same year that the survey was carried out, among these 8,354 blood lead observations were matched to a parcel with surveyed housing conditions. An additional 1,178 observations were excluded because one or more observations of housing conditions were missing, and 578 observations were excluded because the associated home was built after exterior lead paint was banned in 1977. The final study sample included 6,589 observations of pediatric blood lead levels and the conditions of their reported residence. [49]

The study sample selection process is illustrated in **Figure S1**, found in this supplement.

Methods S2: Calculating the Lead Risk Index (LRI)

This section describes how to calculate the Lead Risk index for several example homes.

$$LRI_i = \sum_{j=1}^{5} era_i * condition_{ij} \tag{1}$$

In this equation *i* is the house in question and *j* is the housing category. Beginning with the data in the table below, the first step is to convert to numerical values.

Home (i)	Year of Home Construction	Windows and Doors	Exterior Paint	Porch	Roof	Foundation and Walls
11	1948 (Before 1952)	Severely Deteriorated	Substandard	Substandard	Seriously Deteriorated	Substandard
22	1926 (Before 1952)	Seriously Deteriorated	Severely Deteriorated	Good	Substandard	Good
33	1973 (1952 – 1977)	Good	Severely Deteriorated	Good	Seriously Deteriorated	Substandard
44	1956 (1952 – 1977)	Good	Seriously Deteriorated	Substandard	Excellent	Good
55	2005 (After – 1977)	Good	Good	Good	Substandard	Good

The next step is to calculate the Lead Risk Index.
For Home 1 the LRI is calculated as (2*3) + (2*1) + (2*1) + (2*2) + (2*1) = 16.
For Home 2 the LRI is calculated as (2*2) + (2*3) + (2*0) + (2*1) + (2*0) = 12.
…

Home (i)	Year of Home Construction	Windows and Doors	Exterior Paint	Porch	Roof	Foundation and Walls	LRI
11	2	3	1	1	2	0	16
22	2	2	3	0	1	0	12
33	1	0	3	0	2	1	6
44	1	0	2	1	0	0	3
55	0	0	0	0	1	0	0

Methods S3: Interpreting the Lasso Model

The lasso coefficient reported in Table 2 can be converted into a likelihood. The likelihood that the test subject will have a blood lead level over 3.5 µg/dL when each explanatory variable is equal to zero can be found by using the following formula:

$$P = e^{\beta_0}/\left(1 + e^{\beta_0}\right)$$

The log odds lasso estimate for the intercept is -1.909, thus the likelihood that a child has a blood lead level over 3.5 µg/dL is 12.9% ($e^{-1.909}/(1 + e^{-1.909})$). To interpret the log odds contrast LRI, we add a summation operation to capture the cumulative structure of the dose-response ratio. We can use the following formula:

$$P = e^{\Sigma_0^i(\beta_i)}/\left(1 + e^{\Sigma_0^i(\beta_i)}\right)$$

Thus, the likelihood that a child living in a home with a LRI of 2 has a bll over 3.5 µg/dL is 16.8% ($e^{(-1.909+0.3109)}/\left(1 + e^{(-1.909+0.3109)}\right)$), and the likelihood that a child living in a home with a LRI of 4 has a bll over 3.5 µg/dL is 18.7% ($e^{(-1.909+0.3109+0.1289)}/\left(1 + e^{(-1.909+0.3109+0.1289)}\right)$), and so forth.

Table S1: Characteristics of Blood Lead Data

	Number of Encounters	Percent of Total Encounters	Geometric Mean (sd) BLL in µg/dL*
**Sex**			
Male	3365	51	2.3 (2.1)
Female	3203	49	2.2 (2.1)
Unknown	21	0.3	4.2 (2.1)
**Test Type**			
Venous	4093	62	2.2 (2.2)
Capillary	2015	31	2.6 (1.9)
Unknown	481	7	2.4 (1.9)
**Age in Months**			
Up to 17	1050	16	2.1 (2.0)
18 - 36	2552	39	2.5 (2.2)
37 - 72	2987	46	2.3 (2.1)
**Year of Test**			
2000	848	13	4.2 (2.2)
2001	748	11	3.4 (2.0)
2002	182	3	3.3 (2.0)
2003	53	0.8	3.1 (2.2)
2004	100	2	1.9 (1.9)
2005	52	0.8	1.7 (1.9)
2006	687	10	2.2 (2.0)
2007	792	12	2.1 (2.1)
2008	1386	21	1.8 (1.9)
2009	150	2	2.0 (1.9)
2010	766	11	1.6 (1.7)
2011	222	3	1.7 (1.6)
2012	43	0.7	2.0 (1.8)
2013	560	9	1.7 (1.8)

*Geometric mean calculated suppressing 0 values for BLL. n = 6,510

Table S2: Characteristics of Housing Data

Variable	Rating	Count
Roof		
	Severely Deteriorated	41
	Seriously Deteriorated	509
	Substandard	2551
	Excellent or Good	4066
Foundation and Walls		
	Severely Deteriorated	15
	Seriously Deteriorated	111
	Substandard	568
	Excellent or Good	6473
Windows and Doors		
	Severely Deteriorated	28
	Seriously Deteriorated	94
	Substandard	939
	Excellent or Good	6106
Porch		
	Severely Deteriorated	10
	Seriously Deteriorated	198
	Substandard	1517
	Excellent or Good	5442
Exterior Paint		
	Severely Deteriorated	64
	Seriously Deteriorated	311
	Substandard	1967
	Excellent or Good	4825
Era of Home Construction		
	Pre-1952	5392
	1952-1976	1197
	Post-1977	578

Table S3: Housing Location by Community District

Community District	Count
Old Northeast	2297
East Side	1889
Brush Creek South	806
East Meyer	639
Blue Valley	413
Greater Downtown	377
Hickman Mills	332
Midtown	107
Chouteau	104
Bannister West	41
Brookside	30
Antioch	29
Line Creek Valley	18
Red Bridge East	15
Waldo	14
Grandview Triangle	12
Center	9
Blue Ridge	8
Swope Park	6
Ward Parkway	6
Little Blue Valley	6
Briarcliff	4
Red Bridge West	4
Gashland	1

Figure S1: Study Selection Process

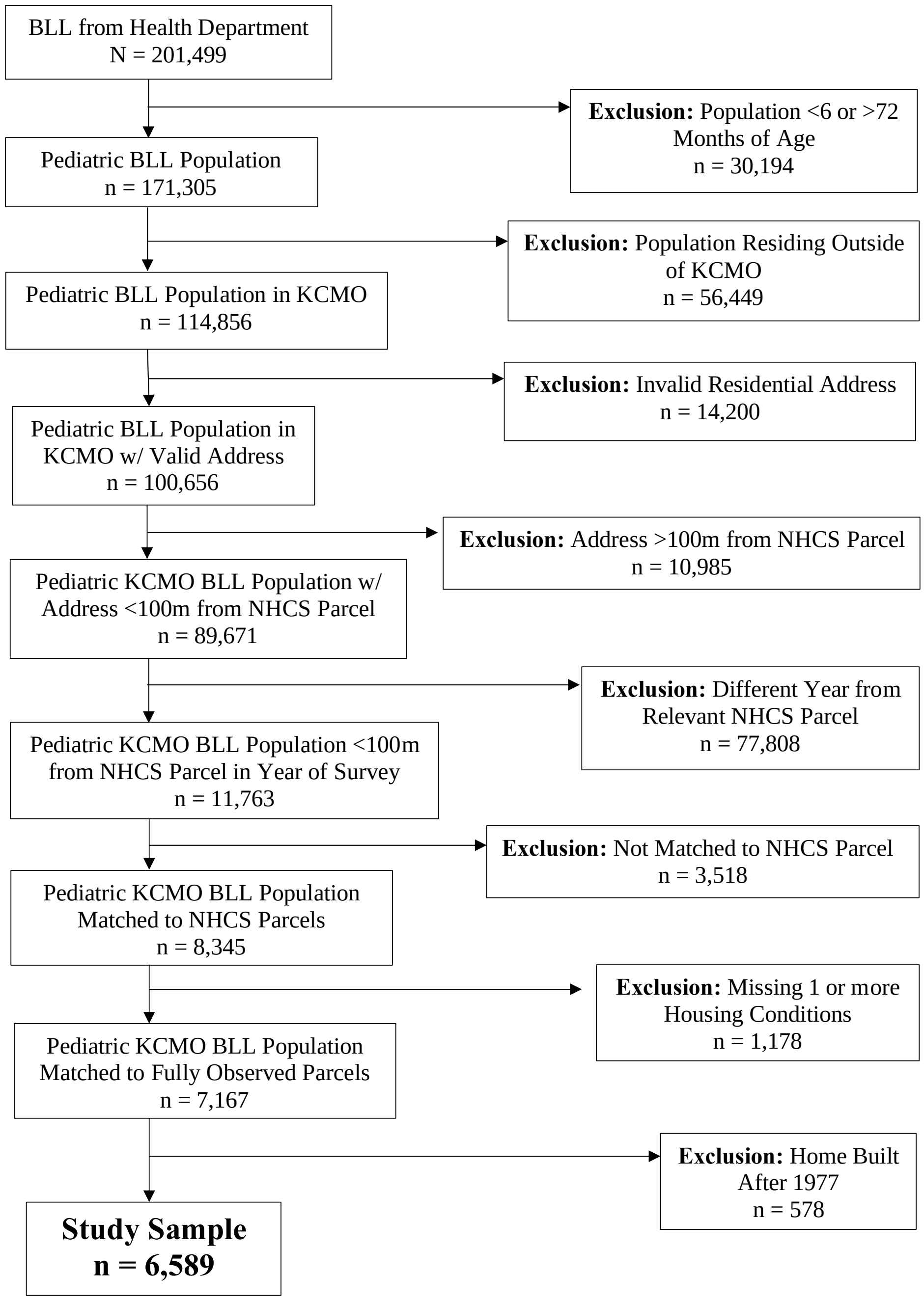

(N. J. Wilson et al. 2023)[49]

Figure S2: Variable Selection Using Lasso Regression

This figure depicts variable selection through lasso regression. In eFigure 2a, red dots denote the penalty coefficient (λ), which, as the value falls, is associated with more included variables. The two dotted lines refer to the λ which minimizes the binomial deviance (left), and the λ one standard deviation above the optimal value (right). In eFigure 2b, each curve matches the track of a single covariate coefficient as the λ parameter falls and more variables are included. Nine covariates are selected in this model, with the optimal λ of 0.00032.

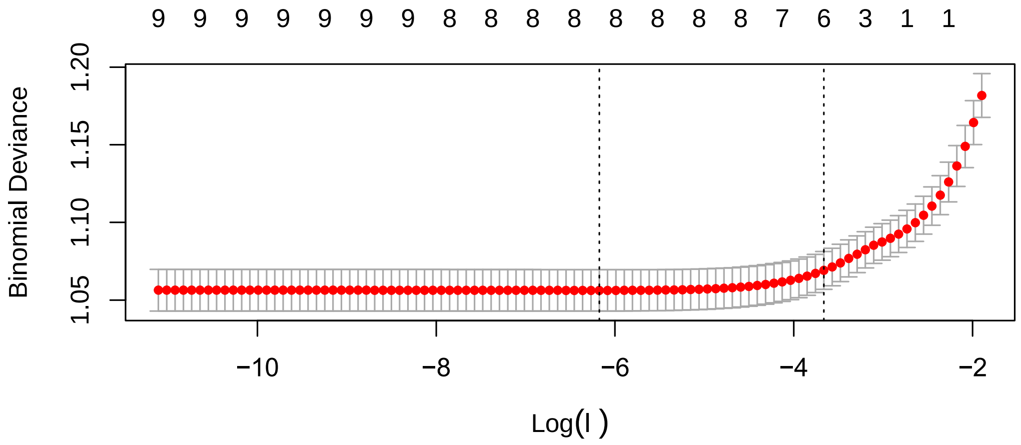

Figure S2A: The optimal penalty coefficient (λ = 0.00032) in the Lasso regression was identified with the minimum criterion

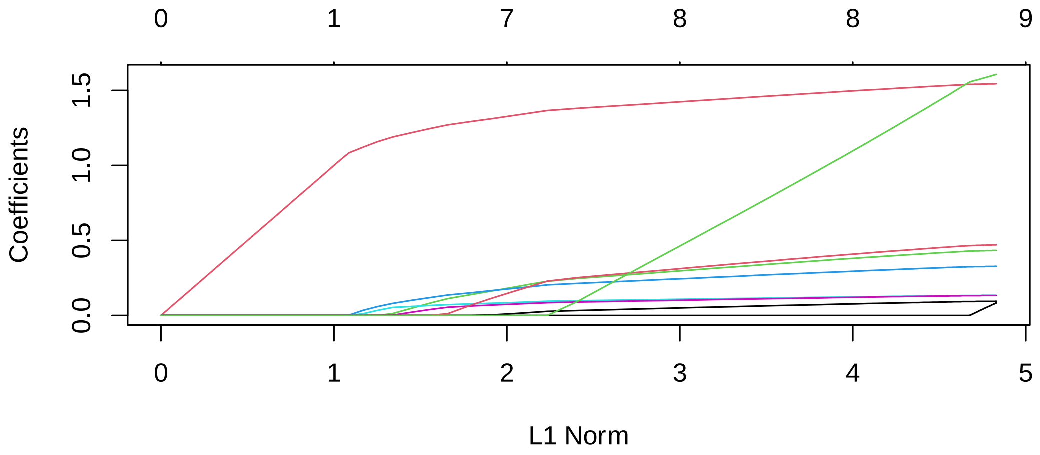

Figure S2B: Lasso coefficient of 9 variables.
